# Changes in Performance of More Than 1000 Minimally Invasive Liver Resections

**DOI:** 10.1001/jamasurg.2020.2623

**Published:** 2020-08-26

**Authors:** Forat Swaid, Iswanto Sucandy, Samer Tohme, James W. Marsh, David L. Bartlett, Allan Tsung, David A. Geller

**Affiliations:** 1 Division of Hepatobiliary and Pancreatic Surgery, Department of Surgery, University of Pittsburgh Medical Center, Pittsburgh, Pennsylvania; 2 Department of Surgery, Advent Health, Tampa, Florida; 3 Department of Surgery, West Virginia University, Morgantown; 4 Department of Surgery, The Ohio State University, Columbus

## Abstract

This cohort study examines changes in laparoscopic liver resection procedures in more than 1000 patients in a single center over a period of 17 years.

Laparoscopic liver resection (LLR) is gaining in popularity. The purpose of this study is to report our performance with minimally invasive liver resections.

## Methods

We report our experience with LLRs in 1062 patients at a single center from calendar years 2001 to 2017. This study received expedited approval from the University of Pittsburgh institutional review board. The approval included waivers of the Health Insurance Portability and Accountability Act and the requirement of documented consent because the study used data previously deidentified. We divided performance into 3 periods, calendar years 2001 through 2007, 2008 through 2012, and 2013 through 2017. We evaluated 5 perioperative outcomes (operating room time, transfusions, use of pure laparoscopic approach, complications, and conversions). The operations were done by 1 of 4 senior liver surgeons (A.T., D.L.B., J.W.M., and D.A.G.) and a hepatopancreatobiliary surgical fellow. Data analysis was completed from January 2018 to March 2018 with Stata version 14 (StataCorp). The statistical significance threshold was set at *P* < .05.

## Results

A total of 1062 patients underwent LLR, including 203 in 2001 through 2007, 426 in 2008 through 2012, and 433 in 2013 through 2017. There were 664 female patients (62.5%) and 398 male patients (37.5%), with a mean age of 58 years (range, 17-94 years), a mean body mass index of 29 (range, 16-61; calculated as weight in kilograms divided by height in meters squared), and an American Society of Anesthesiologists mean (SD) score of 2.6 (0.6). The approach was purely laparoscopic in 724 patients (68.2%), hand-assisted in 134 patients (12.6%), hybrid in 130 patients (12.2%), and robotic in 74 patients (7.0%). Laparoscopic major hepatectomy (right or left lobectomy) was done in 91 of 1062 cases (8.5%), and this did not change across study periods. The indication for resection was a malignant condition in 413 of 1062 cases (38.9%) (hepatocellular carcinoma, 157 [38.0%]; metastatic colorectal cancer, 154 [37.3%]; intrahepatic cholangiocarcinoma, 26 [6.3%]; neuroendocrine tumors, 22 [5.3%]; other, 58 [14.0%]) and symptomatic benign lesions in other cases. Operating room time (mean [SD] times: period 1, 213 [95] minutes; period 2, 195 [87] minutes; period 3, 139 [62] minutes; *P* < .001), packed red blood cell transfusion (period 1, 10 [4.9%]; period 2, 20 [4.7%]; period 3, 4 [0.9%]; *P* = .001), use of a purely laparoscopic approach (period 1, 91 [44.8%]; period 2, 290 [68.1%]; period 3, 302 [69.7%]; *P* < .001), and postoperative complications (period 1, 31 [15.2%]; period 2, 23 [5.4%]; period 3, 23 [5.3%]; *P* < .001) all statistically improved over time (Table). The percentage of laparoscopic or total liver resections (period 1, 203/727 [27.9%]; period 2, 426/1263 [33.7%]; period 3, 433/1371 [31.6%]; *P* = .01) and the presence of background cirrhosis (period 1, 5 [2.4%]; period 2, 10 [2.3%]; period 3, 12 [2.7%]; *P* = .002) increased over time. Simultaneous resection of other organs was low (<3%) and did not change over time. Seventy-seven patients (7.2%) developed complications (cardiopulmonary complication, 22 patients; bile leak, 6; postoperative bleeding, 6; intra-abdominal abscess, 5; thromboembolism, 6; and ileus, 8). The unplanned open conversion rate was 2.5% and did not change across periods. The median hospital length of stay was 2 (range, 1-3) days. Thirty-day and 90-day mortality rates were 2 of 1062 patients (0.2%) and 4 of 1062 patients (0.4%), respectively.

**Table.** Perioperative Parameters by Study Period

Clinical parameter	%			*P* value^a^
	Period 1, 2001-2007 (n = 203)	Period 2, 2008-2012 (n = 426)	Period 3, 2013-2017 (n = 433)	
Operating room time, mean (SD), min	213 (95)	195 (87)	139 (62)	<.001
Transfusions	10 (4.9)	20 (4.7)	4 (0.9)	.001
Purely laparoscopic operations	91 (44.8)	290 (68.1)	302 (69.7)	<.001
Complications				<.001
Conversion to open technique	31 (15.2)	23 (5.4)	23 (5.3)	.82
Background cirrhosis	5 (2.4)	10 (2.3)	12 (2.7)	.002
Laparoscopic cases among all liver resections, No./total No. (%)	203/727 (27.9)	426/1263 (33.7)	433/1371 (31.6)	.01

^a^ Period 1 vs period 2 or 3.

## Discussion

Surgical performance for LLR improved and perioperative morbidity decreased with greater experience. To our knowledge, this is one of the largest single-center series of LLR reported worldwide. Looking at LLR in more than 9500 patients, a meta-analysis^1^ showed comparable mortality and significantly fewer complications, transfusions, blood loss, and hospital stays in LLR vs open liver resection.

The common learning curve parameters are operating room time, conversions, blood loss, and morbidity.^2^ Most laparoscopic learning curve studies report on 1 of these parameters but not others. Performance and learning curves can vary with the degree of difficulty. The learning curve is a moving target and is different in the self-taught era vs the master-apprentice era.

In assessing the learning curve for laparoscopic minor hepatectomy, Vigano et al^3^ reported that conversion rate, operating room time, blood loss, use of the Pringle maneuver, and morbidity improved over 3 periods. When examining laparoscopic major hepatectomy in 173 patients, Nomi et al^4^ identified 3 phases (at 45, 30, and 98 cases) in the learning curve, using cumulative sum chart (CUSUM) analysis for operating time. Operating room time, pedicle clamping, blood loss, and conversions all improved in phase 3 vs phase 1. In 159 cases, van der Poel et al^5^ reported a conversion rate of 11% and a learning curve of 55 cases for laparoscopic hemihepatectomies. When looking at performance in 150 consecutive LLRs, Villani et al^6^ reported 5 groups of 30 consecutive cases per group. Operative complexity for laparoscopic major hepatectomy increased from 3% to 23% (in group 1 vs group 5). Complications decreased from 20% to 3% (in group 1 vs 2) but increased as more complex procedures were performed (in group 2 vs 5).

Based on our data and analysis of other published experiences,^3,4,5,6^ the evolution of LLR learning can be divided into 3 phases defined by 45 and 70 cases (Figure). The learning curve parameters may have periods of improvement and regression as more difficult cases are done until mastery is achieved. The goal is for training programs to shorten the learning curve and improve performance compared with surgeons who are self-taught.

**Figure.**
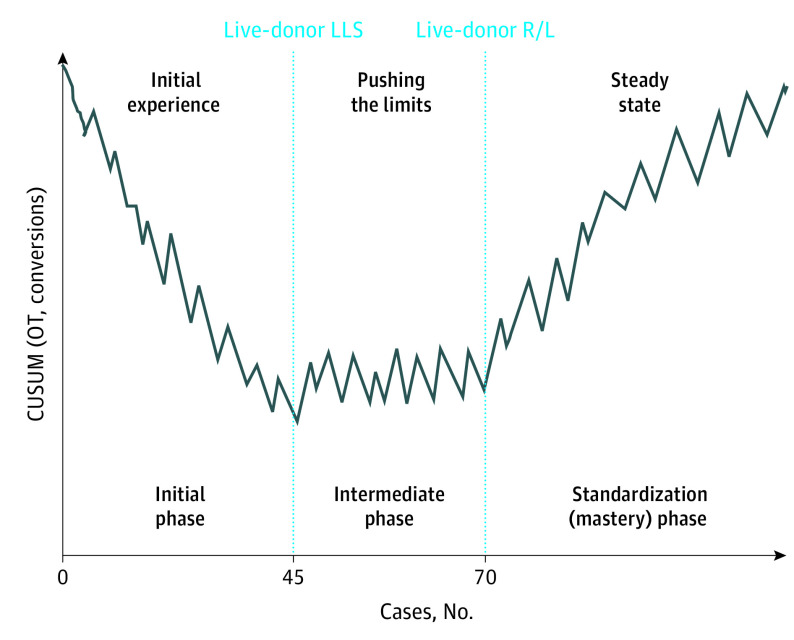
Three Phases in the Evolution of Learning for Laparoscopic Liver Resection The initial phase often includes minor cases, left lateral sectionectomy (LLS), and the left lobe. The intermediate phase is associated with major, difficult segments and the right lobe. The standardization (mastery) phase is characterized by augmented liver reality and 3-dimensional, 4K, or 8K techniques. CUSUM indicates cumulative sum chart; OT, operating times; R/L, right or left lobe.
